# A video intervention reduces racial bias in a representative sample of US adults: A brain as predictor study

**DOI:** 10.1371/journal.pone.0339057

**Published:** 2026-02-19

**Authors:** Yilong Wang, Paul J. Zak

**Affiliations:** 1 Center for Neuroeconomics Studies, Claremont Graduate University, Claremont, California, United States of America; 2 Peter F. Drucker and Masatoshi Ito Graduate School of Management, Claremont Graduate University, Claremont, California, United States of America; Teikyo University - Hachioji Campus: Teikyo Daigaku - Hachioji Campus, JAPAN

## Abstract

Biased attitudes and behaviors towards racial minorities in the US are pervasive, enduring, and detrimental. We tested whether a video illustrating the negative effects of racial bias towards African-Americans would influence short-term and medium-term attitudes and behaviors towards this group. In Experiment 1, a high-impact video was identified by measuring neurologic Immersion in the laboratory (N = 62). Experiment 2 then recruited a representative sample of US adults (N = 1097) to assess the video’s impact on attitudes and behaviors towards African-Americans relative to participants who watched a neutral control video. A two-week follow up study was also done to determine if effects of the treatment video persisted. At baseline, young participants, men, and Republicans, had the statistically highest attitudinal bias towards African-Americans. We found that the treatment video improved average self-reported attitudes towards Black Americans by 11% (p < 0.001; Cohen’s d = 0.26) and generosity in a hypothetical money sharing task with a Black male by 104% (p = 0.016; Cohen’s d = 0.22) compared to controls. These effects were primarily driven by a reduction in attitudinal biases by participants with the highest basal bias. We also showed that these effects were mediated by an increase in positive affect due to the video. Both treatment effects persisted after the washout period indicating that high neurologic Immersion videos may be an effective way to reduce out-group biases at scale.

## Introduction

Out-group bias is pervasive [1–5]. The amount of bias varies by the strength of in-group membership and the perceived differences between groups [6,7]. In-groups are favored because of the increased likelihood of future interactions compared to those from out-groups. Nonhuman animals also favor in-groups, showing its evolutionary basis [8–10]. Yet, avoiding or derogating out-groups is detrimental in many ways—for example, it limits the number and quality of social connections, reduces economic trading partners, and leads to hiring less qualified employees [11–18].

In-group bias often influences people’s choices. In money sharing experiments, participants tend to transfer more and are willing to accept less from individuals they identify as members of their in-group [19–21]. Helping those in need and outright avoidance also show in-group biases, though many conditional factors influence such behaviors [22–25]. Some teams that include in- and out-group members are less productive while others are more productive, showing the subtlety of how people interact in groups [26–29]. When groups are defined by race, biased decisions may be driven by the ease of identifying emotional states from members of one’s racial group [30].

Ethnicity and race are common ways people assign group membership because they are easy to observe [31,32]. In-group biases based on race may have biological foundations as shown in neuroimaging and genetic studies, and even children show preferences for their in-groups [33–37]. Even given these predilections, race can be “erased” when other meaningful group indicators are used to replace presumed out-group indicators. Such replacements are effective even with minimal group identifiers [38–40]. Another successful approach to reduce out-group bias introduces people from different groups to each other, an approach known as “contact theory” [41,42]. Variants of contact theory that reduce bias include indirect contact [38,43], extended contact [44] and imagined contact [45,46].

“Erasing race” is a laudable goal, yet nearly all of the approaches seeking to accomplish this require in-person interventions, making them time- and cost-intensive. For example, cross-race contact using virtual reality (VR) experiences with racially identifiable avatars reduces bias, but VR headsets are not in common use and remain expensive [47]. A less expensive approach, the use of videos discussing the negative impacts of racial biases, have produced mixed effects [48,49]. If we seek to have a race-blind society, we must find methods to reduce out-group bias at scale. This is the focus of the present research.

The nearly universal access to video content in the US suggests that videos could be a scalable bias-reduction intervention. Rather than guess if a video would effectively reduce bias, we created a library of publicly available videos and, in Experiment 1, measured them for neurologic Immersion to select the one that was most likely to influence attitudes and behaviors toward an out-group. The measurement of neurologic responses has been used to successfully predict aggregate outcomes, an approach called “brain as predictor” [50–53]. Once a high Immersion video was identified, in Experiment 2 this video was sent to a representative sample of US adults and their attitudes and behaviors towards a racial out-group were quantified and compared to a control sample. We hypothesized that the treatment video would reduce outgroup bias using both attitudinal measures and a behavioral task. By measuring both attitudes towards an out-group as well as cooperative behavior with a member of this group, we were able to examine the consistency of hypothesized anti-bias effects. Participants in the treatment condition were also invited to a two-week follow-up assessment to determine if the influence of the video was sustained.

## Methods

### Experiment 1

This study was designed to rigorously and transparently identify a video describing the detrimental effects of out-group biases that might reduce default attitudes and behaviors. Rather than ask participants which videos they believed would be effective, which has poor predictive accuracy [54–56], we measured neurologic Immersion of videos in order to rank them for expected influence on attitudes and behaviors.

Immersion is a combination of neuroelectrical signals that appears to reflect the brain’s valuation network for social-emotional experiences [57–60]. Immersion was identified during 15 years of research that searched for combinations of neural signals that would accurately and consistently predict individual and group behaviors after a message or experience [53]. The components of Immersion include attention (associated with frontal dopamine binding) and emotional resonance (traced to the activity of oxytocin in the brainstem and subgenual cortex [61]). These signals were then convolved to maximize the predictive accuracy of behaviors. For example, Immersion predicts hit songs three months in advance with 97% accuracy, mood and energy with 98% accuracy [59], as well as identifies which videos people choose to watch [58], and how much customers will spend while shopping [62]. Peak Immersion experiences produce strong emotional responses, which is why they influence attitudes and behaviors [60,63].

### Participants

Sixty-four participants (76% female) were recruited from the Claremont Colleges and surrounding community using our standing subject pool (age: M = 22.64, SD = 7.56). Participants were US residents who spoke fluent English. This study was approved by the Institutional Review Board of Claremont Graduate University (#4295) and followed the guidelines of the Declaration of Helsinki. All participants gave written informed consent prior to inclusion. No personally identifiable information was collected, and data were anonymized using an alpha-numeric code. Two participants’ data were excluded due to device connection issues for a final sample size of 62.

### Dates

This study was done from 2/1/2023–4/1/2023.

### Procedure

**Fig 1:**
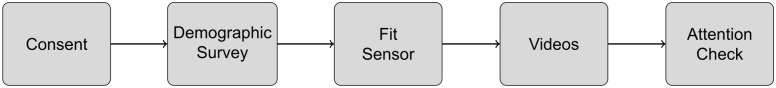
Experiment 1 timeline.

After consent, participants completed surveys on demographics. Participants were then seated and fitted with photoplethysmography (PPG) sensors (Rhythm +, Scosche Industries, Oxnard, CA) placed on their forearms. Next, groups of 3–6 participants were informed that they would watch several videos. Videos were displayed on a large monitor in a medium-sized lab. Participants were told they would answer questions about the videos to ensure they attended to the content; all participants passed the attention check (Fig 1). The study lasted 45 minutes and participants were paid $15 for their time.

### Neurophysiology

PPG data were captured by a Bluetooth hub that relayed signals to a commercial neuroscience platform (Immersion Neuroscience, Henderson, NV). The Immersion Neuroscience platform applies algorithms to variations in cardiac responses that capture the activity of the cranial nerves associated with central neuroelectrical Immersion signals at 1 Hz [59,61]. Following previous analyses, we used the Immersion time-series data to derive a second measure of neurologic responses, peak Immersion (PEAK [58,61]). PEAK is defined as


$${\text{PEAK}}_{\text{ij}}=\;\int_{t=\text{0}}^{T}(n_{ijt}>M_{i})d_{t},$$

where *n* corresponds to Immersion for participant *i* watching video *j*, that runs from time *t = 0* to time *T*, and *M*_*i*_ is the median level of Immersion plus one standard deviation across all videos watched by participant *i*. The Immersion time series has a sine wave pattern as the brain has refractory periods after a significant metabolic response resulting in a strong tendency to return to baseline [53]. PEAK sums the Immersion values that exceed the threshold *M* which often shows more variation than average Immersion during a stimulus [63].

### Videos

A library of five videos, each of moderate length, was built from publicly available online content from YouTube that discussed the negative effects of racial biases (Length: M = 2m44sec; Range: 2m11sec - 3m 16sec). Our focus was on attitudes towards African-Americans because of the current salience of an actual or perceived bias against this group in the US [64,65]. We used a modestly sized video library because the stimuli would be ranked for neurologic effects by exposing them to participants rather than having the researchers “guess” which video would have the largest effect.

### Statistical analysis

Pairwise t-tests and one-way ANOVAs were used to evaluate neurological responses to the videos in order to identify the one that had the highest likelihood of influencing attitudes and behaviors.

### Experiment 2

This study was designed to test if the video chosen in Experiment 1 would reduce out-group bias towards African-Americans in a representative sample of US adults. An additional goal was to identify for which demographic groups bias was reduced, if any. In order to establish baseline bias without inducing physiologic arousal, a video control (VC; 2m21sec) was sent to a sample of participants showing natural scenes and with relaxing music as in previous research [52,66]. The remainder of the sample viewed the video treatment (VT, 3m15sec).

### Participants

A nationally representative sample of 1,200 adult participants was recruited from the Qualtrics panel (Qualtrics, Inc., Provo, UT). This study was approved by the Institutional Review Board of Claremont Graduate University (#4517), followed the guidelines of the Declaration of Helsinki, and all participants gave written informed consent prior to inclusion. The data were anonymized by assigning an alpha-numeric code to each participant. Participants were randomly assigned to watch VT or VC, and the final sample (N = 1097) are those participants who passed an attention check during the study (VT: n = 802; VC: n = 295). A large sample size was used to ensure ecological validity and obviates the need for a statistical power calculation.

### Dates

Data collection was done from 7/1/2024–9/1/2024.

### Surveys

After providing informed consent, participants completed a demographic questionnaire. Next, they provided answers to the Positive and Negative Affect Schedule (PANAS; [67]) prior to viewing the video and immediately after it to measure the change in affect. Participants were then invited to answer questions about their perceptions of African-Americans using the psychometrically-valid Attitudes Towards Blacks survey (ATB; [68,69]). The ATB consists of 20 questions on a 7-point Likert scale. Half of the questions have positive valence (ATB_p_) and half have negative valence (ATB_n_).

In order to make the ATB measure easier to interpret, we constructed a variable called attitude bias (AB):


$$AB=\left(ATB_{n}-ATB_{p}+\text{7}\text{0}\right)\;*\;\frac{\text{1}\text{0}\text{0}}{\text{1}\text{4}\text{0}}$$

This normalizes the ATB so that AB = 0 indicates no bias, and the maximum bias is 100. This scaling affects the absolute values of the bias measure but not its statistical properties. The analysis will also report values for ATB_p_ and ATB_n_.

**Fig 2:**
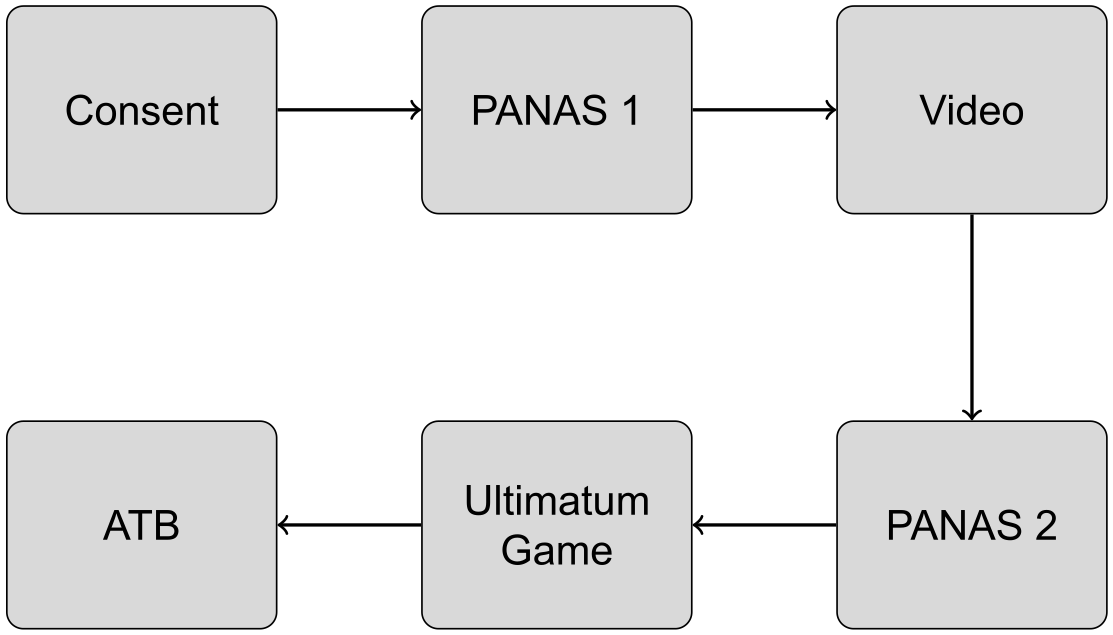
The timeline of Experiment 2.

After completing the ATB, participants were instructed in, and completed, a behavioral task described below. Note that we will use both African-American and Black throughout this paper to indicate American residents who self-identify as having African heritage because both terms are in common usage and the ATB uses the term “Black” [70]. Fig 2 shows the sequence of tasks participants completed in Experiment 2.

### Behavioral task

In order to quantify out-group bias, participants were instructed in, and made a choice, in a cognitively simple share-the-money task from experimental economics called the ultimatum game (UG; [71–73]). Decisions in the UG require using theory of mind to infer others’ intentions and will be described below [74]. The UG has been used to assess generosity or stinginess toward those of a different racial group [19,75].

The UG randomly assigns participants to the roles of decision-maker 1 (DM1) or decision-maker 2 (DM2) and both are identically instructed in the task. After instruction, DM1 is prompted to propose a split of $10 to DM2. Both DMs know that DM2 can either accept or reject the proposed split. If the proposal is accepted the money will be transferred, but if the proposal is rejected both DMs earn zero. The instructions included numerical examples showing choices and payoffs. We used the strategy method to heighten theory of mind by asking participants to specify their choices as DM1 and the minimum amount they would accept as DM2 [76].

Three measures of cooperative behaviors were obtained from the UG: the proposed monetary offer by DM1, the DM2 minimum acceptable offer, and generosity defined as the DM1 proposal minus the DM2 minimum [72].

A 50% ($5) proposal would be a fair split of $10, while proposals less than 50% are viewed as unfair [77]. Higher offers and lower minimum acceptance levels indicate a desire to cooperate with another, while generosity measures the degree to which a participant will benefit another at a cost to oneself [78]. Participants were informed they would make hypothetical choices and these have been shown to be an effective measure of cooperative intent [76,79]. Participants were randomly selected to make decisions in the UG in which the other DM was identified with a typical male white name (Mike) or a typical African-American male name (Demetrius). The UG instructions are shown in the Supporting Information.

### Statistical analysis

Baseline biases were established by using values obtained from participants who watched the control video (VC). T-tests of mean differences from the baseline average were used to identify demographic groups with the highest bias. The impact of the treatment video (VT) was initially established by comparing mean values in the ATB and UG to control values. Demographic groups with high baseline biases where then examined to determine if the intervention affected biases as measured by the ATB and UG. Next, a linear regression was estimated to confirm the robustness of the bivariate results with and without the inclusion of control variables. The truncation of the ATB values necessitated correcting the regression errors using Tobit estimation [80,81]. In addition, a mediation model was estimated in order to examine if the treatment influenced attitudes and behaviors by changing affect as shown in related research [52]. Finally, the persistence of the treatment was tested by within-subjects comparisons after a two-week washout period. There is no theory on the length of the washout period to sustain the behavioral effects of a video stimulus so two weeks seemed reasonable [82]. Only a subset of treatment participants completed the follow-up survey and as a result, a correction was utilized to remove the possibility of a selection bias [83,84].

## Results

### Experiment 1: Video selection

There was no difference in average Immersion across videos (one-way ANOVA F = 0.94, p > .05). However, there was a difference in PEAK among the videos (one-way ANOVA F = 7.87, p < 0.001). Pairwise t-tests identified the video with the highest PEAK value (M_PEAK_ = 3224.32, SD_PEAK_ = 1358.69) and it was significantly greater than the video with the second-highest PEAK (M = 2572.05, t = 2.69, p = 0.008; all PEAK data are shown in the Supporting Information in Appendix 1). The video with the highest peak Immersion was an animated depiction of the childhood of Dr. Ronald McNair, the second African-American NASA astronaut. Dr. McNair died in the 1986 Challenger disaster and the video was narrated by Ronald’s older brother Carl. The story discusses the racism Dr. McNair faced as a child in South Carolina and how he overcame this to earn a PhD in physics from MIT. The video was recorded by StoryCorps and the References include a link to the video which we recommend readers view [85].

### Experiment 2: Baseline bias

Mean baseline values for the ATB survey were derived from participants who watched the control video. Table 1 reports the baseline bias in the control group.

**Table 1:** Baseline bias from the control group is shown for the Attitudes Towards Blacks, positive (ATBp), Attitudes Towards Blacks, negative (ATBn), Attitude Bias as defined above (AB), and monetary transfer choices made in the ultimatum game (UG) by decision-maker 1 (DM1), decision-maker 2 (DM2), and overall monetary generosity in the UG (the difference between DM1 and DM2 transfers). These data show that there is a moderate level of bias against blacks in the US population.

Variable	Mean	Std. Dev.	Min	Max
ATBp	53.60	12.23	10	70
ATBn	33.12	14.58	10	70
AB	35.37	15.83	7.14	92.86
DM1	5.56	1.71	0	10
DM2	4.75	2.03	0	10
Generosity	0.81	2.00	−5	10

Next, we examined demographic categories to identify which groups had statistically larger biases on the ATB survey. Men had a 25.8% higher bias towards African-Americans compared women as the result of both lower positive views and higher negative views (ΔAB_male_ = 8.09, t = 4.54, p < 0.001; ΔATBp_male_ = −4.36, t = −3.11, p = 0.002; ΔATBn_male_ = 6.98, t = 4.22, p < 0.001). Republicans also had 14.0% more bias towards African-Americans compared to those from all other self-identified political groups. As with men, Republicans had more negative views of Blacks and trended toward lower positive views (ΔAB_Repub_ = 4.67, t = 2.51, p = 0.013; ΔATBp_Repub_ = −2.82, t = −1.95, p = 0.052; ΔATBn_Repub_ = 3.72, t = 2.17, p = 0.03). Younger participants (age: 18–43) had 14.0% more average bias toward African-Americans due to lower positive views and higher negative attitudes (ΔAB_Young_ = 4.65, t = 2.54, p = 0.012; ΔATBp_Young_ = −3.17, t = −2.24, p = 0.026; ΔATBn_Young_ = 3.34, t = 1.98, p = 0.049).

### Baseline behavioral bias

Control participants who made decisions in the UG with a presumed black male had an average generosity of $0.49 which is no different than the average generosity shown towards a presumed white male participant (White M = $0.81, t = −1.19, p = 0.24). Neither men nor Republicans exhibited less generosity toward Blacks in the UG (Male: M_W_ = 0.77, M_B_ = 0.21, t = −1.37, p = 0.17; Repub: M_W_ = 0.90, M_B_ = 0.66, t = −0.56, p = 0.58). The correlation between being Republican and male in this sample was not significant (r = 0.052 p = 0.086). Similar to the AB results, bias toward Blacks in generosity decreased linearly with age (r = −0.20, t = −2.82, p = 0.005).

### Treatment Effects

**Fig 3:**
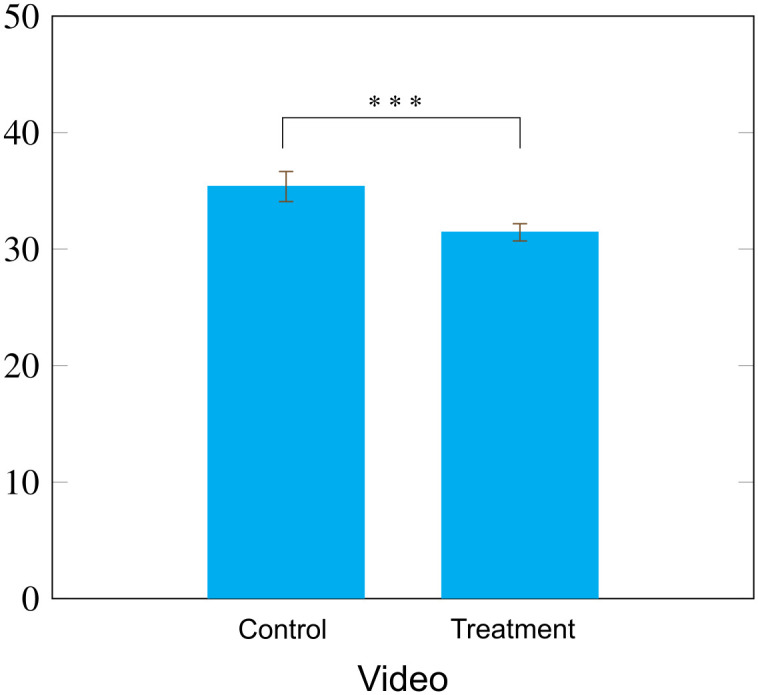
Attitude bias towards African-Americans was 11% lower for those who watched the treatment video compared to those in the control group (p < 0.001). Bars are standard errors. *** = p < .001.

The treatment video decreased participants’ average bias towards Blacks by 11% compared to controls (M_T_ = 31.44, M_C_ = 35.37; t = −3.78, p < 0.001; Cohen’s d = 0.26; CI: [−5.97,-1.89]; Fig 3). The treatment increased both positive perceptions of Blacks (ΔATB_p_ = 2.24, t = 2.90, p = 0.004; Cohen’s d = 0.20; CI: [0.72,3.76]) and reduced negative attitudes (ΔATBn = −3.26, t = 3.36, p < 0.001; Cohen’s d = 0.23; CI: [−5.16,-1.35]).

**Fig 4:**
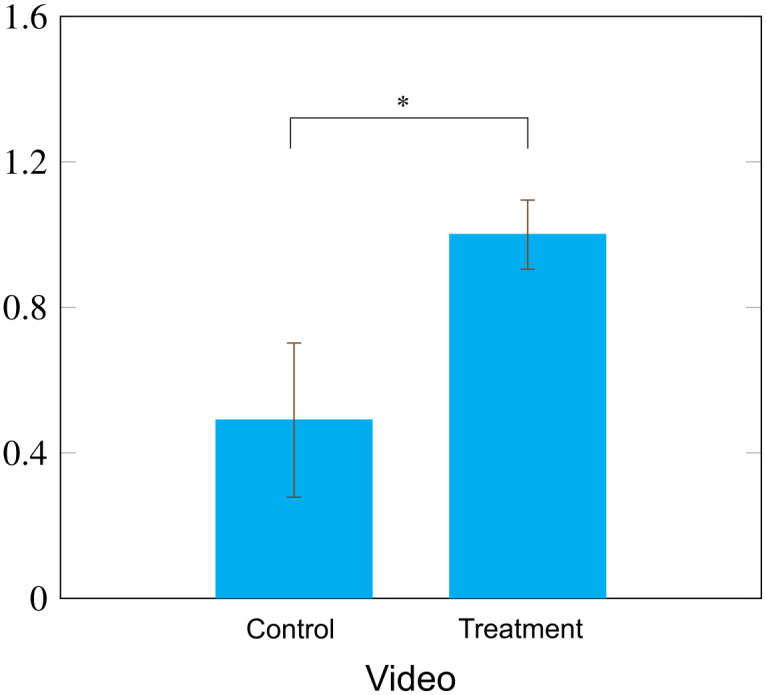
Generosity towards African-Americans was 104% higher for those in the treatment group matched to a person with a stereotypical African-American name compared controls with the same matching (p = .0016). Bars are standard errors. * = p < .05.

Confirming the decrease in reported bias in the ATB, the treatment increased average generosity in the UG towards a person with an African-American name by 104% compared to control African-American generosity (M_T_ = 1.00, M_C_ = 0.49; t = 2.41, p = 0.016; Cohen’s d = 0.22; CI: [0.09,0.93]; Fig 4). This was the result of being willing to accept less as DM2 after watching the video (M_T_ = 4.51, M_C_ = 5.17; t = −3.48, p < 0.001; Cohen’s d = 0.32; CI: [−1.03,-0.29]) rather than a difference in DM1 proposed splits (M_T_ = 5.51, M_C_ = 5.66; t = −0.89, p = 0.37; Cohen’s d = 0.08; CI: [−0.47,0.18]). The treatment did not influence generosity in UG decisions when matched with a stereotypical white name (M_T_ = 1.11, M_C_ = 0.81; t = 1.29, p = 0.20; Cohen’s d = 0.13; CI:[−0.16,0.75]). The effect of the treatment in reducing bias in the AB was significantly correlated with generosity in the UG (r = −0.16; p < 0.001, CI:[−0.22,-0.10]).

### Treatment effects for demographic segments

The treatment reduced the male attitude bias towards Blacks by 12.8% (M_T_ = 34.45, M_C_ = 39.49, t = −3.40, p < 0.001). Indeed, men who viewed the treatment video had higher positive views of Blacks and lower negative views (ATBp: M_T_ = 54.54, M_C_ = 51.39, p = 0.0064; ATBn: M_T_ = 32.78, M_C_ = 36.67, t = −2.70, p = 0.0071). Behaviorally, men in the treatment group were 319% more generous in the UG than controls (M_T_ = 0.88, M_C_ = 0.21, t = 2.11, p = 0.036). As with the overall results, men in the treatment condition were willing to accept less when sharing money (DM2: M_T_ = 4.72, M_C_ = 5.55, t = −2.94, p = 0.0035). The treatment had no effect on men making decisions in the UG matched to a white name (M_T_ = 0.73, M_C_ = 0.77, t = 0.13, p = 0.90).

The effect of the treatment on Republicans mirrored the effects on men. Reported bias by treatment Republicans was 8.8% lower compared to Republican controls (M_T_ = 34.77, M_C_ = 38.14, t = −2.12, p = 0.035). This was driven by a reduction in negative attitudes (M_T_ = 32.44, M_C_ = 35.33, t = −1.97, p = 0.049) rather than an increase in positive attitudes (M_T_ = 53.76, M_C_ = 51.93, t = 1.46, p = 0.14). The treatment did not increase generosity towards African-Americans in the UG among Republicans (M_T_ = 1.01, M_C_ = 0.66, t = 1.05, p = 0.29), though it did reduce the DM2 cooperation threshold (M_Repub_ = 4.54, t = −1.97, p = 0.05).

Among younger participants, the treatment video reduced bias towards Blacks by 9.5% (M_T_ = 34.14, M_C_ = 37.73, t = −2.39, p = 0.017). This was due to an increase in positive attitudes (M_T_ = 54.44, M_C_ = 51.99, t = 2.06, p = 0.042) rather than a decrease in negative attitudes (M_T_ = 32.23, M_C_ = 34.82, t = −1.74, p = 0.083). The treatment did not change young participants’ generosity towards Blacks in the UG (M_T_ = 0.68, M_C_ = 0.66, t = 0.07, p = 0.94).

### Robustness

The bivariate findings were tested for robustness by estimating an ordinary least-squares regression that included three independent variables that were found to be significantly associated with bias in the AB (male, Republican, age). Consistent with our previous findings, the OLS estimation with AB as the dependent variable showed that treatment participants had lower bias levels compared to controls (b_T_ = −3.17, t = −3.20, p = 0.0014), while at the same time male sex, Republican, and age were significant and had the same signs as in the bivariate analyses (b_Male_ = 6.98, t = 5.56, p < 0.001; b_Repub_ = 7.68, t = 5.75, p < 0.001; b_age_ = −0.21, t = −3.20, p < 0.001).

Re-estimating the OLS equation using generosity as the dependent variable confirmed that the treatment increased generosity (b_T_ = 0.45, t = 2.13, p = 0.033). Contrary to the bivariate findings, the treatment increased generosity for older participants (b_age_ = 0.011, t = 2.11, p = 0.035), but did not support its influence on men (b = −0.24, t = −1.33, p = 0.18) or Republicans (b = 0.036, t = 0.19, p = 0.85).

### Mediation effects

**Fig 5:**
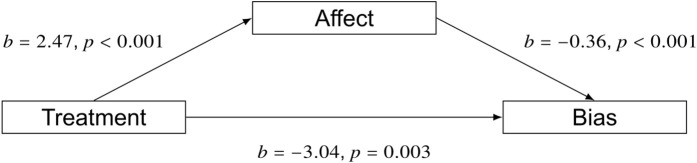
A mediation model shows the treatment video decreased bias by raising positive affect (b = −0.89, p < 0.001) as well as directly (b = −3.04, p = 0.003).

**Fig 6:**
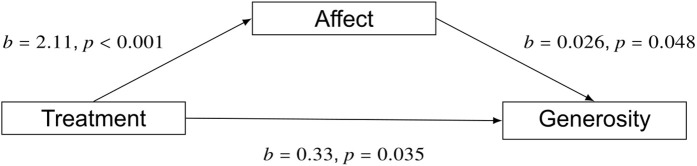
Estimating a mediation model for generosity shows that the treatment increased positive affect and thereby increased generosity (b = 0.058, p = 0.029) as well as the treatment having a direct effect on generosity (0.33, p = 0.035).

We tested the role of affect in influencing attitudes and behaviors by estimating a mediation model using the change in positive affect (∆PA) as the mediator. The estimation showed that the treatment reduced AB through its influence on positive affect as well as directly (Direct: b −3.04, p = 0.003; Indirect: b = −0.89, p < 0.001, R^2^ = .25; Fig 5). Estimating another mediation model for generosity also showed a significant influence of positive affect as well as a direct effect of the video treatment (Direct: b = 0.33, p = 0.035; Indirect: b = 0.058, p = 0.029. Fig 6).

### Persistence

We repeated the ATB survey and UG task after a two-week washout period to assess if the treatment effects would persist (n = 97). We first tested whether the demographics of participants in the follow up survey matched those of the first wave sample in order to ensure any persistence results were not due to a selection bias [86]. This analysis showed that the wave 2 sample was less educated than those in wave 1 (College degree: M1 = 0.49, M2 = 0.37; *X*(1)=4.55, p = 0.033). We subsequently removed the effect of a college education on answers to the ATB and decisions in the UG by running a linear regression. Paired t-tests for the variables with the selection effect removed showed that the reduction in bias was sustained during the washout period in both the ATB and UG (∆AB = 1.78, p = 0.17; ∆UG = −0.33, p = 0.31). This was confirmed by the unchanged level of positive and negative attitudes in ATB (∆ATBp = −0.82, p = 0.47; ∆ATBn = 1.67, p = 0.26) and in UG decisions (∆DM1 = 0.082, p = 0.72; ∆DM2 = 0.41, p = 0.17).

## Discussion

Contact with out-group members has been considered the gold standard in reducing bias [87]. Yet, there are many ways to reduce out-group biases. For example, when personal contact with in-group members induces oxytocin release from the brain, out-group bias in a money sharing task is eliminated [21]. Since direct contact approaches do not scale, we investigated the use of a video to reduce negative attitudes and behaviors towards a US out-group, Black Americans.

There are several noteworthy innovations in our approach and findings. First, rather than guess or use inaccurate self-reported “liking” of videos, Experiment 1 developed a library of videos and ranked them using a neurophysiologic measure that has been shown to accurately predict post-message behaviors [52,53,88] and has 98% concordance with self-reported mood [59,63]. The video was a short, animated biography of an outstanding African-American who grew up in the racially-divided southern US, earned a PhD in physics, and was also an astronaut. The video did not overtly discuss reducing racism but showed how one person overcame racist attitudes.

Our results indicate that peak Immersion messaging is a systematic and potentially replicable way to influence attitudes and behaviors at scale. This approach could be used, for example, by government agencies or nonprofits, that seek to reduce biased attitudes and behaviors by creating videos and measuring them for Immersion before sharing them with the public. Moreover, there are large size effects when measuring neurologic Immersion so that stimuli need only be measured for 35–70 people to reach statistical power > .90 [89]. One goal of this research was to develop and test a methodology that is straightforward to apply and can be easily deployed.

The payoff from identifying a high Immersion video in Experiment 1 was the significant size effects found in Experiment 2. Average bias toward Blacks, as measured by AB, decreased 11% while average generosity towards Blacks in the UG more than doubled when compared to controls. Importantly, both of these effects persisted after a two-week washout period. A meta-analysis of 515 contact studies found that intergroup contact reduced out-group bias with a mean correlation between the degree of contact and bias of r = −.21 [42]. Our finding of an 11% reduction in attitudinal bias using a single video compares well to the reduced bias from in-person contact. The 104% increase in monetary generosity shows an even larger effect of the treatment on behavior.

Perhaps our most compelling finding is the persistence of the treatment effects after a two-week washout period. We first removed the selection bias among participants in the second wave of data collection and then demonstrated treatment persistence for both the attitudinal and behavioral measures of bias. If reducing biases is a societal goal, then medium- and long-term effects must be assessed for efficacy, yet this is rarely done [90,91]. Future research should extend the two-week washout period used here as well as should evaluate the effects of multiple high Immersion video interventions viewed over the course of weeks or months to determine their cumulative impact on attitudes and behaviors for a year or longer.

We traced the psychological mechanism producing the improvement in attitudes as well as greater generosity towards Blacks to an increase in positive affect by estimating a mediation model. Consistent with our findings, positive affect is known to decrease out-group biases and racism [92–94]. This result enables scholars and practitioners who extend our work to focus not only on identifying high Immersion videos to reduce bias, but videos that increase positive affect. An increase in positive affect appeared to reinforce the neurologic impact of the treatment video and thereby reduce biases. While many high Immersion videos are associated with positive mood states, this is not always the case and thus is important to assess [52,53,58]. It is also worth noting that the video did not influence attitudes or behaviors that did not involve Black Americans, showing the treatment had a narrow focus as expected.

The location that a stimulus is experienced and the medium of presentation also appears to matter [95]. Our approach sent the treatment video to participants to watch anywhere they chose and where distractions were likely, increasing the ecological validity of the findings compared to a laboratory study [96,97]. Future research should also consider augmented and virtual reality stimuli as a potentially more effective way to influence attitudes and behaviors. For example, a patient experience video presented in virtual reality produced greater peak Immersion in nursing students than the same video presented in a standard two-dimensional format and influenced a prosocial behavior by raising empathic concern [89]. Virtual and augmented reality videos about the detrimental effects of racial biases may be similarly potent.

There are several areas of concern and some reasons for hope in our findings. The first concern is that while the degree of bias toward Black Americans using the attitudinal measure is relatively low on average, the most bias was found, in order, for younger participants (aged 18–43), men, and Republicans. The treatment reduced attitudinal bias towards African Americans in all three groups from between 9–13%, but this was not enough to bring any of these demographic segments back to the sample average bias. Our finding of greater bias in younger adults and men is consistent with research using different methodologies [98,99]. Moreover, this finding is unlikely to be statistical noise since the treatment reduced bias in all three groups. Videos targeted at these demographic groups could be produced to reduce bias where it is highest using the methodology described herein.

Another point of concern is that the treatment, while increasing monetary generosity towards a person with an African-American name in the UG on average, did not affect generosity in two of the three groups reporting the highest attitudinal bias. Among men, the treatment increased generosity in the money-sharing task by 319% compared to controls. But, the treatment did not affect generosity for younger participants or Republicans. As a result, the treatment effects for these groups as measured by the ATB should be seen as provisional until they are confirmed using behavioral measures of bias. Our findings should also be replicated to examine other out-group biases, including towards those of various ethnicities, religious affiliations, and countries-of-origin in order to develop a robust methodology to reduce biases.

Out-group bias is pervasive due in part to the likelihood of repeat interactions and future opportunities to cooperate with in-groups [100]. While race is an obvious group indicator, it is also one that is easy to replace [21,32,40]. This suggests the approach here can be applied to reduce a variety of out-group biases, for example, towards those with different sexual orientations, nationalities, genders, or religions, which may make a more harmonious world.

## Supporting information

Appendix 1 Average peak Immersion values for all five videos in Experiment 1. (DOCX)
