# Supplementary material for: A video intervention reduces racial bias in a representative sample of US adults: A brain as predictor study
Source: PLoS One. 2026 Feb 19;21(2):e0339057. doi: 10.1371/journal.pone.0339057 (PMC12919826; doi:10.1371/journal.pone.0339057)
Supplement: Appendix 1 — (DOCX) [file pone.0339057.s001.docx]

**Appendix: Behavioral Task (Ultimatum Game)**

*DM1*.

Imagine that you must decide how to split $10 between yourself and *NAME*.

*NAME* knows that $10 is the full amount being divided.

You may offer any amount of the $10 (i.e. $0, $1, $2, $3, $4, $5, $6, $7, $8, $9, $10) to *NAME* (keeping for yourself what remains).

But, if *NAME* rejects the amount you offer, then both of you lose the $10 altogether and end up with nothing.

How much will you offer *NAME* if you want your offer to be accepted?

$ amount to offer *NAME*:

*DM2*.

Now reverse the roles:

Imagine that *NAME* gets to decide how to split $10 between you both.

The same rule applies, where if you reject *NAME*’s offer, you both will receive nothing.

What is the smallest amount you would accept from *NAME*?

Minimum acceptable amount from *NAME*:

**Appendix: Figure A1**

Average peak Immersion values for all five videos in Experiment 1.

[Fig A1 here]
